# The Oligopoly of Academic Publishers in the Digital Era

**DOI:** 10.1371/journal.pone.0127502

**Published:** 2015-06-10

**Authors:** Vincent Larivière, Stefanie Haustein, Philippe Mongeon

**Affiliations:** 1 École de bibliothéconomie et des sciences de l’information, Université de Montréal, C.P. 6128, Succ. Centre-Ville, Montréal, QC. H3C 3J7, Canada; 2 Observatoire des Sciences et des Technologies (OST), Centre Interuniversitaire de Recherche sur la Science et la Technologie (CIRST), Université du Québec à Montréal, CP 8888, Succ. Centre-Ville, Montréal, QC. H3C 3P8, Canada; Katholieke Universiteit Leuven, BELGIUM

## Abstract

The consolidation of the scientific publishing industry has been the topic of much debate within and outside the scientific community, especially in relation to major publishers’ high profit margins. However, the share of scientific output published in the journals of these major publishers, as well as its evolution over time and across various disciplines, has not yet been analyzed. This paper provides such analysis, based on 45 million documents indexed in the Web of Science over the period 1973-2013. It shows that in both natural and medical sciences (NMS) and social sciences and humanities (SSH), Reed-Elsevier, Wiley-Blackwell, Springer, and Taylor & Francis increased their share of the published output, especially since the advent of the digital era (mid-1990s). Combined, the top five most prolific publishers account for more than 50% of all papers published in 2013. Disciplines of the social sciences have the highest level of concentration (70% of papers from the top five publishers), while the humanities have remained relatively independent (20% from top five publishers). NMS disciplines are in between, mainly because of the strength of their scientific societies, such as the ACS in chemistry or APS in physics. The paper also examines the migration of journals between small and big publishing houses and explores the effect of publisher change on citation impact. It concludes with a discussion on the economics of scholarly publishing.

## Introduction

This year (2015) marks the 350^th^ anniversary of the creation of scientific journals. Indeed, it was in 1665 that the *Journal des Sçavans* and the *Philosophical Transactions* of the Royal Society of London were first published, in France and in England respectively. They were founded with the intent to advance scientific knowledge by building on colleagues’ results and avoid duplication of results, and established both the principles of scientific priority and peer review. They changed the process of scholarly communication fundamentally, from personal correspondence through letters (which had become “too much for one man to cope with in his daily reading and correspondence”) [1], society meetings, and books to a more structured and regular distribution of scientific advancements. This structured form, combined with a regular and wide dissemination, enabled systematic recording and archiving of scientific knowledge [1–4].

Since the 17^th^ century, the importance of journals for diffusing the results of scientific research has increased considerably. After coexisting alongside correspondence, monographs and treaties—which often took several years to be published—they became, at the beginning of the 19^th^ century, the fastest and most convenient way of disseminating new research results [5–7] and their number grew exponentially [1,8]. During the 20^th^ century they consolidated their position as the main media for diffusing research [6], especially in the natural and medical sciences [9]. Scholarly journals also contributed to the professionalization of scientific activities by delimiting the frontier between popular science and the research front and, as a consequence, increased the level of specialization of research and the formation of disciplines. Interestingly, while the majority of periodicals emerged from scientific societies, a significant proportion were published by commercial ventures as early as in the Victorian era. At that time, these commercial publishing houses proved more efficient in diffusing them than scientific societies [10]. However, prior to World War II, most scholarly journals were still published by scientific societies [11]. Data from the mid-1990s by Tenopir and King [12] suggests an increase of commercial publishers’ share of the output; by then, commercial publishers accounted for 40% of the journal output, while scientific/professional societies accounted for 25% and university presses and educational publishers for 16%. Along these lines, the UK Competition Commission measured various publishers’ shares of ISI-indexed papers for the 1994–1998 period and showed that, over this period, Elsevier accounted for 20% of all papers published [13]. One could expect, however, that these numbers have changed during the shift from print to electronic publishing. Indeed, many authors have discussed the various transformations of the scholarly communication landscape brought by the digital era (see, among others, Borgman [14–15]; Kling and Callahan [16]; Tenopir & King [17]; Odlyzko [18]). However, although the digital format improved access, searchability and navigation within and between journal articles, the form of the scholarly journal was not changed by the digital revolution [16,19]. The PDF became the established format of electronic journal articles, mimicking the print format [20]. What was affected by the digital revolution is the economic aspect of academic publishing and the journal market.

The literature from the late 1990s suggests that the digital era could have had two opposite effects on the publishing industry. As stated by Mackenzie Owen [21], while some authors saw the Web as a potential solution to the serials’ crisis—decreasing library budgets facing large and constant annual increases of journal subscription rates [22,23]—most authors hypothesized that it would actually make the situation worse [24] or, at least, not provide a solution [25,26]. Despite the fact that it is generally believed that the digitalization of knowledge diffusion has led to a higher concentration of scientific literature in the hands of a few major players, no study has analyzed the evolution over time of these major publishers’ share of the scientific output in the various disciplines. This paper aims at providing such analysis, based on all journals indexed in the Web of Science over the 1973–2013 period.

## Methods

This paper uses Thomson Reuters’ Web of Science (WoS)—including the Science Citation Index Expanded, the Social Sciences Citation Index and the Arts and Humanities Citation Index—transformed into a relational database optimized for bibliometric analysis. On the whole, 44,483,425 documents are analyzed for the 1973–2013 period, which include all document types published by various journals. In addition to indexing authors’ names, addresses and cited references, which are the units of analysis typically used in bibliometric studies, the WoS indexes the name, city and country of the publisher of the journal for each issue. Using this information, which changes over time, we are thus able to assign journals and papers to a publisher and see the evolution of journal ownership. One limitation of this source of data is that it does not index all of the world’s scientific periodicals but only those indexed in the WoS, which meet certain quality criteria such as peer review and which are the most cited in their respective disciplines. Hence, this analysis is not based on the entire scientific publication ecosystem but, rather, on the subset of periodicals that are most cited and most visible internationally.

The journal publishing market is a complex and dynamic system, with journals changing publishing houses and publishing houses acquiring or merging with competitors. Although these changes should be reflected in the publisher information provided for each issue, in some cases, the name of the publisher does not change immediately after a merger or an acquisition. Publishers’ activities are often distributed among multiple companies under their control, and over the past 40 years, there have been many mergers and acquisitions involving entire companies or parts of them. We looked at the mergers and acquisitions history of major publishers, based on their number of papers published, in order to identify and associate the companies that came to be under their control, and conversely the companies which they eventually sold. These publishers are the American Chemical Society, American Institute of Physics, American Physical Society, Cambridge University Press, Emerald, IEEE, Institute of Physics, Karger, Nature Publishing Group, Optical Society of America, Oxford University Press, Reed-Elsevier, Royal Society of Chemistry, Sage Publications, Springer, Taylor & Francis, Thieme Publishing Group, Wiley-Blackwell, and Wolters Kluwer. For example, Reed-Elsevier bought Pergamon Press in 1991 but, in the WoS, journals remain associated with Pergamon Press until the year 2000. Hence, we assigned any journal published by Pergamon Press since 1991 to Reed-Elsevier. In the case of partial acquisitions, journals were assigned to the publisher only if at least 51% of the company was under its control. Historical merger and acquisition data up to 2006 was found in the report by Munroe [27]. The data for subsequent years was retrieved from the companies’ profiles in the Lexis Nexis database, as well as in the press releases found on publishers’ websites.

## Results

**Fig 1:**
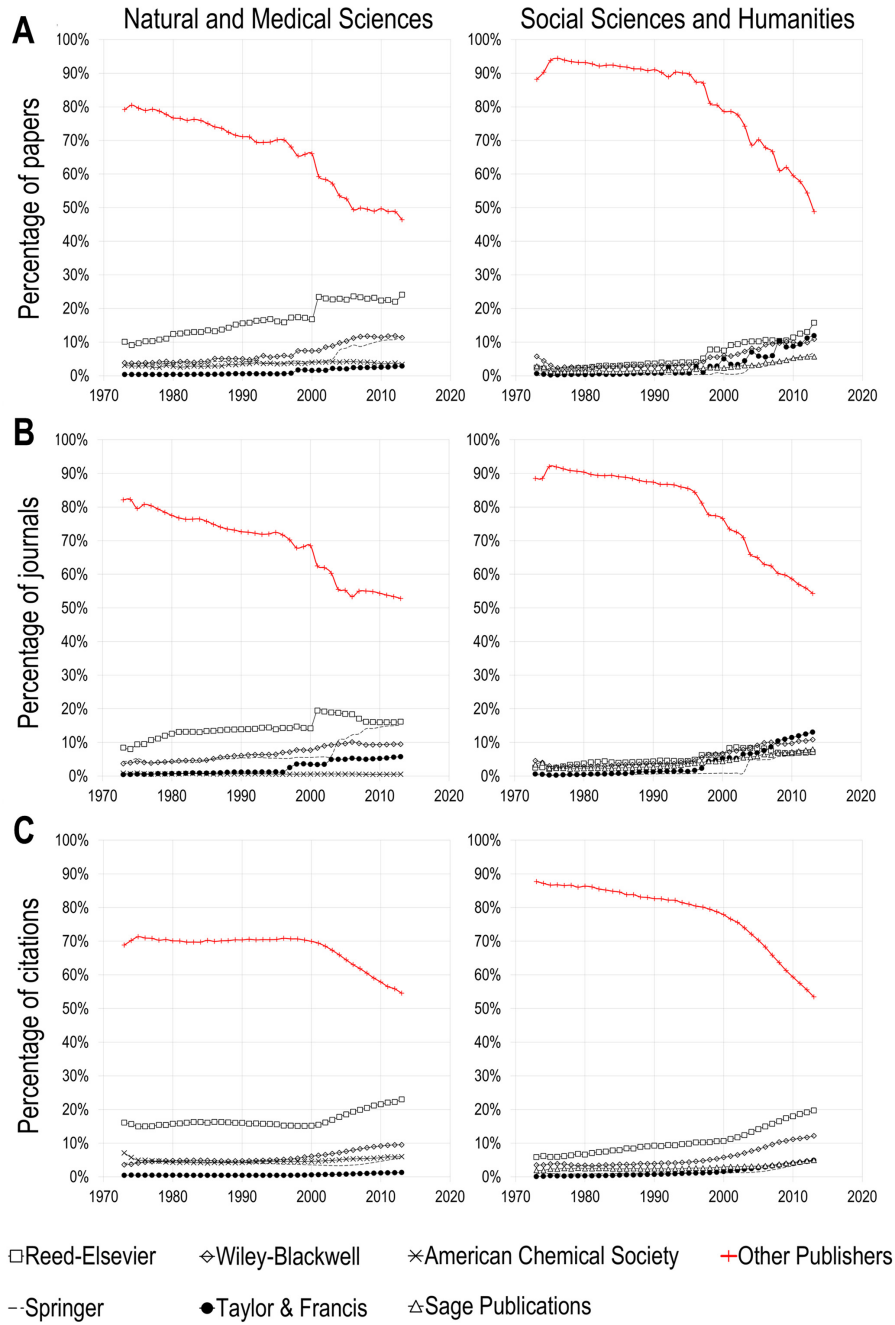
Percentage of Natural and Medical Sciences (left panel) and Social Sciences and Humanities (right panel) papers published by the top 5 publishers, 1973–2013.

Fig 1A presents, for Natural and Medical Sciences (NMS) and Social Sciences and Humanities (NMS), the proportion of papers published by the top five publishers that account for the largest number of papers in 2013, as well as the proportion of papers published in journals others than those of the top five publishers. Fig 1B provide numbers for the proportion of journals published by various publishers, while Fig 1C presents the publishers’ share of citation received. What is striking for both domains is the drop, since the advent of the digital era in the in the mid-1990s, in the proportion of papers, journals and citations that are published/received by journals from publishers *other* than the five major publishers. In both NMS and SSH, Reed-Elsevier, Wiley-Blackwell, Springer, and Taylor & Francis are amongst the top five publishers with the highest number of scientific documents in 2013. While in NMS the American Chemical Society makes it to the top five (in fourth place in 2013), the fifth most prolific publisher in the SSH is Sage Publications. Hence, while all top publishers in SSH are private firms, one of the top publishers in NMS is a scientific society.

In terms of numbers of papers published, the five major publishers in NMS, accounted, in 1973, for little more than 20% of all papers published. This share increased to 30% in 1996, and to 50% in 2006, the level at which it remained until 2013 when it increased again to 53%. In this domain, three publishers account for more than 47% of all papers in 2013: Reed-Elsevier (24.1%; 1.5 fold increase since 1990), Springer (11.9%; 2.9 fold increase), and Wiley-Blackwell (11.3%; 2.2 fold increase). The American Chemical Society (3.4%; 5% decrease) and Taylor & Francis (2.9%; 4.9 fold increase) only account for a small proportion of papers. In the SSH, the concentration increased even more dramatically. Between 1973 and 1990, the five most prolific publishers combined accounted for less than 10% of the published output of the domain, with their share slightly increasing over the period. By the mid-1990s, their share grew to collectively account for 15% of papers. However, since then, this share has increased to more than 51%, meaning that, in 2013, the majority of SSH papers are published by journals that belong to five commercial publishers. Specifically, in 2013, Elsevier accounts for 16.4% of all SSH papers (4.4 fold increase since 1990), Taylor & Francis for 12.4% (16 fold increase), Wiley-Blackwell for 12.1% (3.8 fold increase), Springer for 7.1% (21.3 fold increase), and Sage Publications for 6.4% (4 fold increase). On the whole, for these two broad domains of scholarly knowledge, five publishers account for more than half of today’s published journal output. Very similar trends are observed for journals and citations, although with a less pronounced concentration, especially for citations in NMS which have remained quite stable between 1973 and the late 1990s. For instance, while the top 5 publishers account for 53% (NMS) and 51% (SSH) of papers, their proportion of journals is of 53% (NMS) and 54% (SSH), and of 55% (NMS) and 54% (SSH) when it comes to citations received. This suggests that the top 5 publishers publish a higher number of papers per journal than other publishers not making the top five, and that their papers obtain, on average, a lower scientific impact.

**Fig 2:**
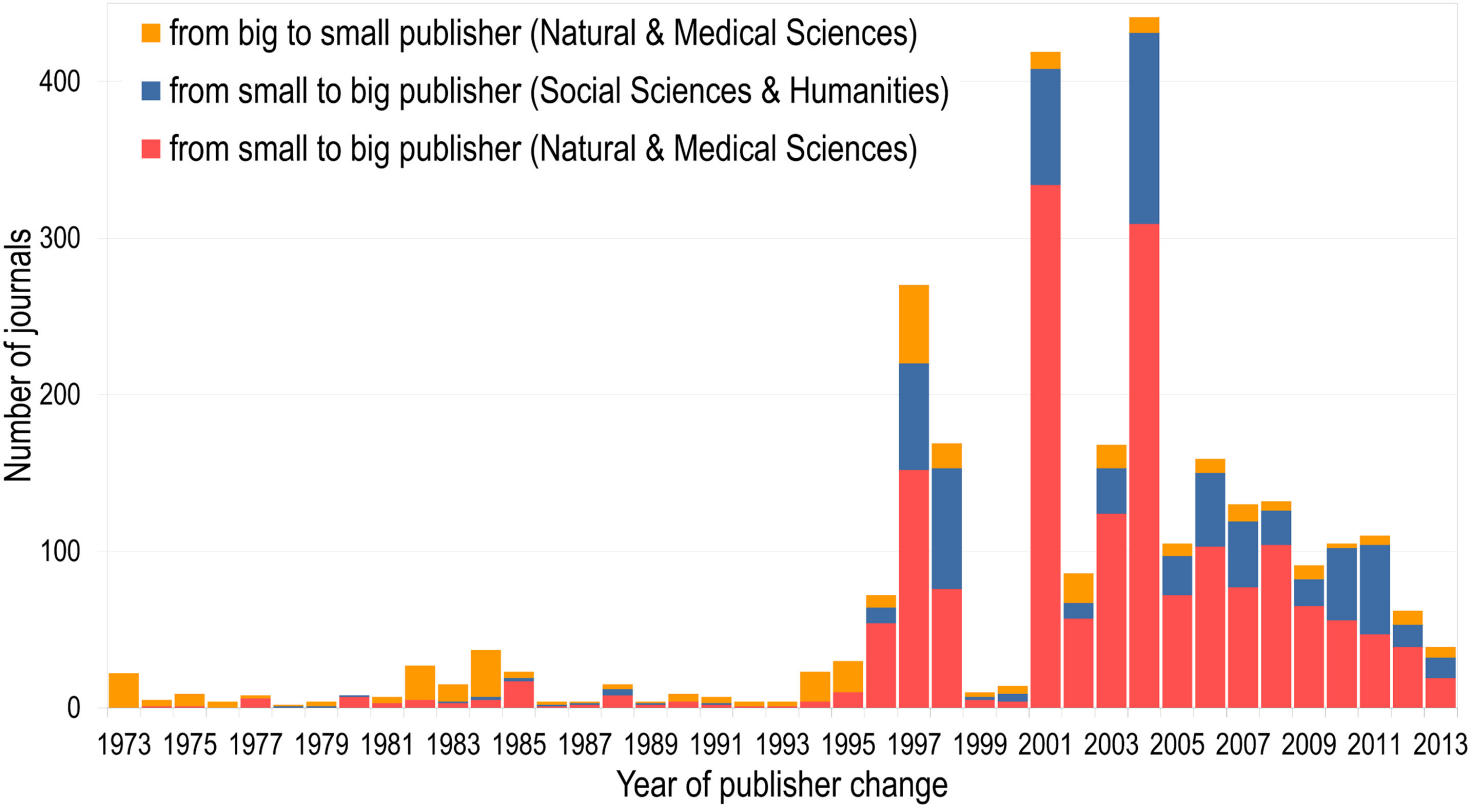
Number of journals changing from small to big publishers, and big to small publishers per year of change in the Natural and Medical Sciences and Social Sciences & Humanities.

The increase in the top publishers’ share of scientific output has two main causes: 1) the creation of new journals and 2) existing journals being acquired by these publishers. Fig 2 presents, for both NMS and SSH, the number of journals over time that changed ownership from small to big publishers—that is, the four publishers with the largest share of published papers in both NMS and SSH—and, for NMS, the number of journals that moved from big to small publishing houses. Since we intend to emphasize developments of the publishing market by publisher type and not single actors, changes among small as well as among big publishers are not shown. It can be seen in both domains that, before 1997, publisher type changes were overall quite rare and the majority consisted of changes from big to small publishers in NMS. Importantly, not a single journal was found to have switched from a big to small publisher in SSH during the entire period of analysis. A first important large wave of journal acquisitions by the big publishers occurred in 1997–1998, when Taylor & Francis acquired several journals from Gordon & Breach Science Publishers, Harwood Academic Publishers, Scandinavian University Press, Carfax Publishing and Routledge. In the same period Reed-Elsevier acquired a few small publishers like Butterworth-Heinemann, Ablex Publications, JAI press, Gauthier-Villars and Expansion Scientifique Française. The next important peak occurred in 2001, and is mainly due to Reed-Elsevier continuing a series of acquisitions, including Academic Press, Churchill Livingstone, Mosby and WB Saunders. Finally, the peak of 2004 is mainly due to the acquisition of Kluwer Academic Publishers by Springer, who had not previously been involved in substantial journal acquisition activities. Wiley-Blackwell’s contribution to the four peaks in Fig 2 was steadier, with the company acquiring an average of 39 journals annually from various publishers during the 2001–2004 period.

**Fig 3:**
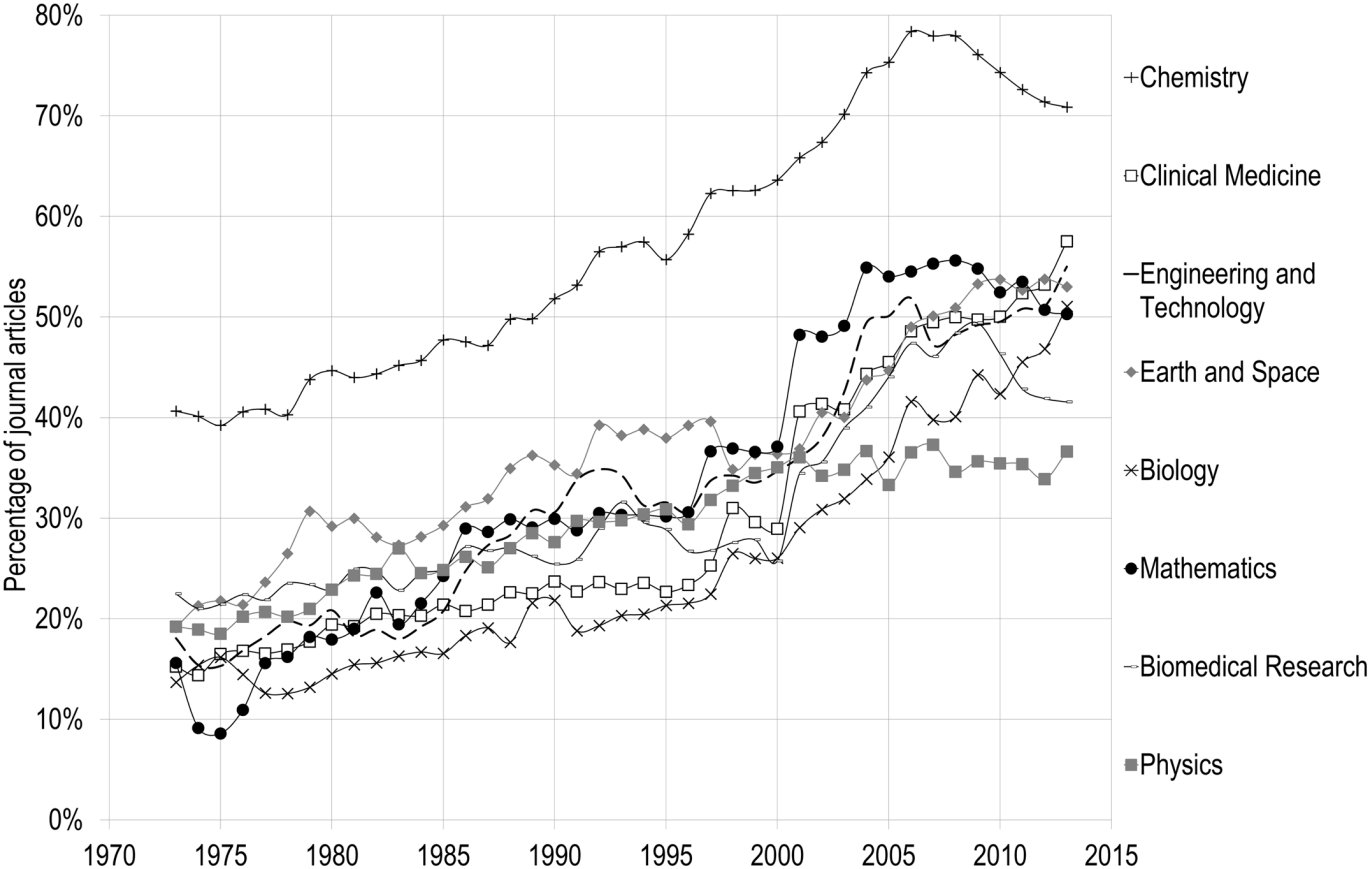
Percentage of papers published by the five major publishers, by discipline in the Natural and Medical Sciences, 1973–2013.

**Fig 4:**
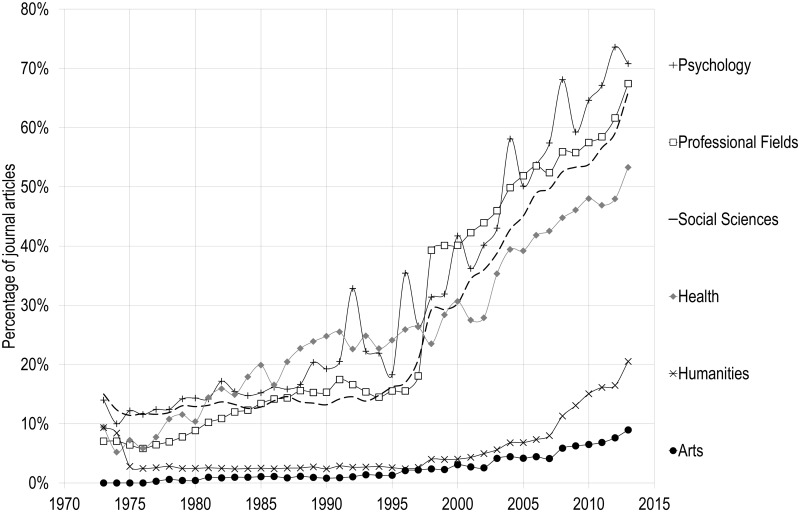
Percentage of papers published by the five major publishers, by discipline of Social Sciences and Humanities, 1973–2013.

**Fig 5:**
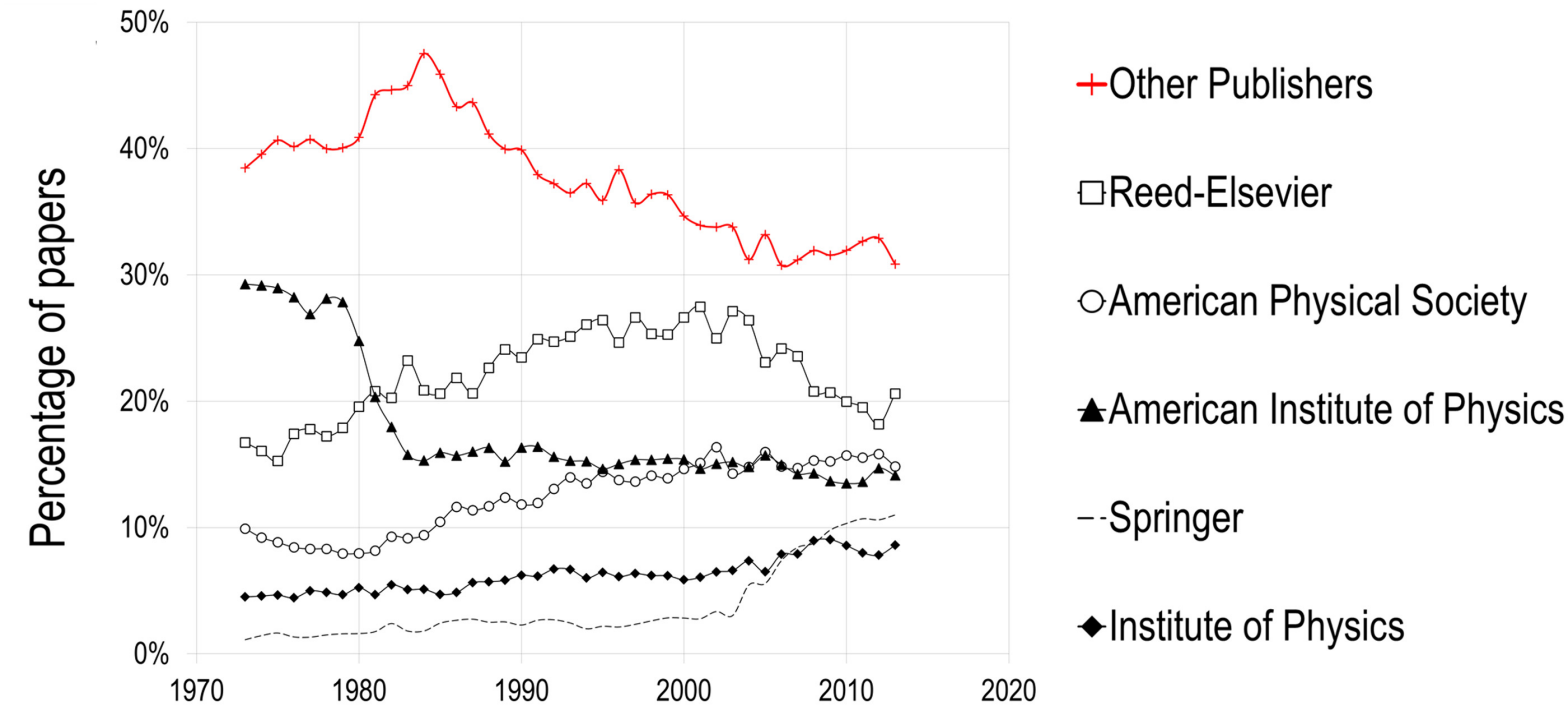
Percentage of papers published by the five major publishers in Physics, 1973–2013.

The share of journal papers published by the five publishers differs amongst the various disciplines in NMS and SSH. Figs 3 and 4 present the evolution of the top five publishers’ share of papers by discipline. Not surprisingly, chemistry has the highest level of concentration, as one of its disciplinary publishers, the ACS, made it to the top five most prolific publishers of NMS. For most disciplines, however, concentration in the top five publishers increased from between 10% and 20% in 1973 to between 42% and 57% in 2013, with a clear change of slope in the mid-1990s. Physics, on the other hand, follows a different pattern: after increasing from 20% in 1973 to 35% in 2000, it has since then remained stable and is subsequently the discipline where the top five publishers account for the lowest proportion of papers published. This lower concentration of papers in big publishers’ journals is mainly due to the strength and size of physics’ scientific societies, whose journals publish an important proportion of scientific papers in the field (Fig 5). In 2013 for instance, journals of the American Physical Society (APS) and of the American Institute of Physics (AIP) each account for 15% of papers, while those of the Institute of Physics (IOP) represent 8% of papers. It is also worth noting that, in physics, Reed-Elsevier’s journals’ share of papers also decreased over the last decade or so, from 28% of papers in 2001 to 21% in 2013. Springer, however, increased its percentage of physics papers from 3% to 11% over the same period. On the whole, the central importance of scientific societies in physics, the presence of arXiv, the central preprint server of physics, astrophysics and mathematics, as well as Open Access agreements such as SCOAP3 (http://scoap3.org/), are likely to make the field less profitable and thus less interesting for commercial publishers. In biomedical research, the share of the top five publishers almost reached 50% in 2009 (49%), but then decreased to 42% in 2013, mainly as a result of the emergence of new publishers, such as the Public Library of Science and its mega-journal PLOS ONE, which publishes more than 30,000 papers per year.

Fig 4 clearly shows that disciplines typically labelled as ‘social sciences’ behave differently from the arts and humanities. For each discipline within the domain of social sciences (psychology, professional fields, social sciences and social aspects of health), there is an unambiguous change in the slope in the mid-1990s: while the top five—in this case, commercial—publishers accounted for percentages between 15% and 22% of the output in 1995, these percentages increased to between 54% and 71% in 2013. The disciplines in social sciences, which includes specialties such as sociology, economics, anthropology, political sciences and urban studies, is quite striking: while the top five publishers accounted for 15% of papers in 1995, this value reached 66% in 2013. Combined, the top three commercial publishers alone—Reed-Elsevier, Taylor & Francis and Wiley-Blackwell—represent almost 50% of all papers in 2013. Psychology follows a similar pattern, with the top five publishers increasing from 17% in 1995 to 71% in 2013.

On the other hand, papers in arts and humanities are still largely dispersed amongst many smaller publishers, with the top five commercial publishers only accounting for 20% of humanities papers and 10% of arts papers in 2013, despite a small increase since the second half of the 1990s. The relatively low cost of journals in those disciplines—a consequence of their lower publication density—might explain the lower share of the major commercial publishers. Also, the transition from print to electronic—a strong argument for journals to convert to commercial publishers—has happened at a much slower pace in those disciplines as the use for recent scientific information is less pressing [28]. Moreover, these disciplines make a much more important use of books [9] and generally rely on local journals [29], all of which are factors that make it much less interesting for big publishers to buy journals or found new ones in the arts and humanities.

**Fig 6:**
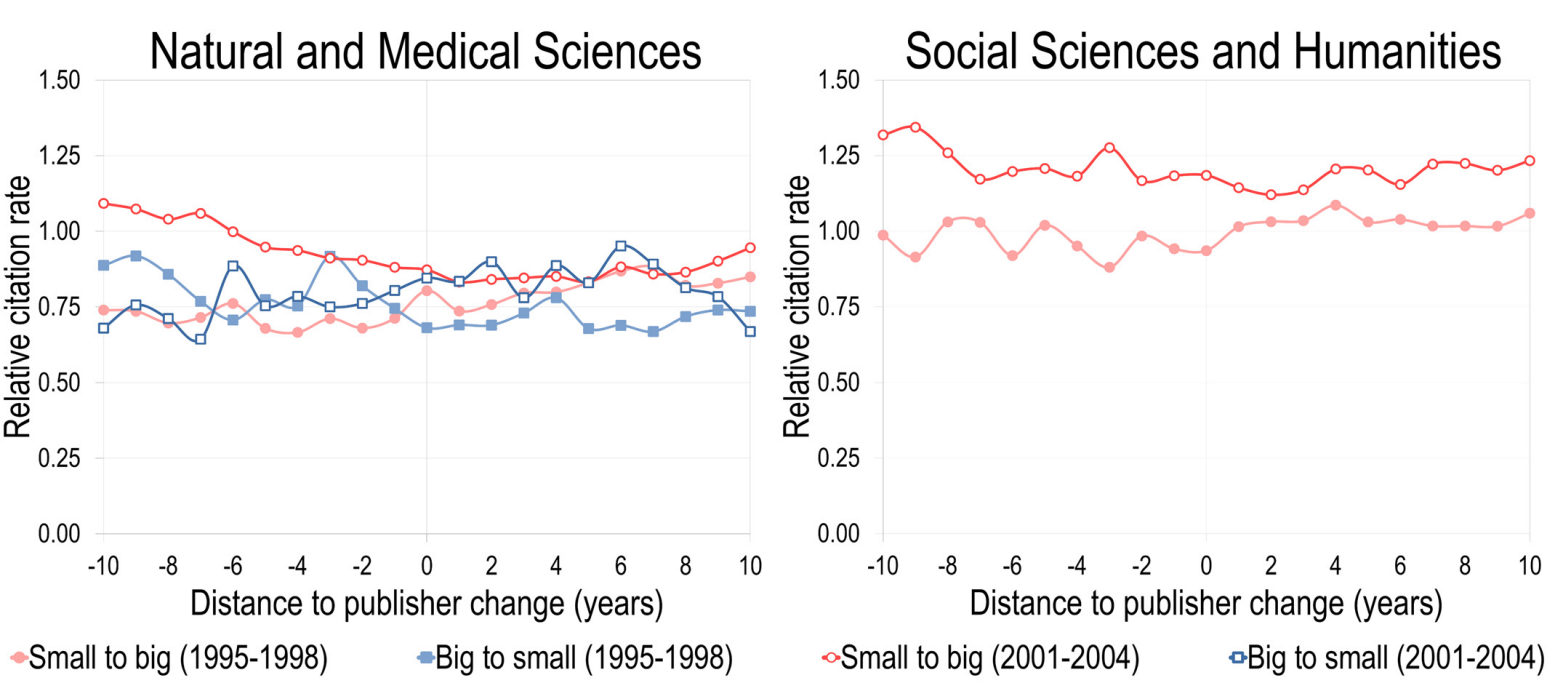
Evolution of the mean relative citation impact of papers, by distance to publisher change, 1995–1998 and 2001–2004.

Fig 6 presents the changes in articles’ relative citation rates for journals that have changed from small to big and big to small publishers (see Fig 1) for the 10 years before and after the transition. We focus on two four-year periods to ensure comparable environments for the publishing market and selected 1995–1998 and 2001–2004, as they were identified as important consolidation phases in Fig 1. More specifically, for NMS, those that have changed from small to big publishers increased their impact slightly following the change. However, while for the 2001–2004 cohort of journals this followed a drop in impact, impact of the 1995–1998 cohort was relatively stable before. For the journals moving from big to small publishers, there is no effect: impact remains similar prior to and after the change. In SSH, no noticeable effect can be observed: changing from a small to a big commercial publisher does not affect papers' citation rates. It is also worth mentioning that, except for journals moving from small to big publishers between 2001 and 2004, the mean impact of papers before and after remained below the world average. It suggests that, on average, journals changing publishers did not produce many high impact papers.

## Discussion and Conclusion

### The effect of scientific societies

On the whole, our results show that the top commercial publishers have benefited from the digital era, as it led to a dramatic increase in the share of scientific literature they published. It has also led to a greater dependence by the scientific community on these publishers. Despite the fact that the two broad domains have both experienced an increase in the concentration of papers in the hands of a few publishers reaching 50% in recent years, a clear distinction was observed between NMS and SSH. In the former group of disciplines, the size of scientific societies—which is a consequence of the size of disciplines in general—managed to keep the literature less dependent on commercial publishers. For example, scientific societies such as the ACS or the APS publish many journals in the specialties of chemistry and physics respectively, for which they successfully managed the shift from print to electronic. On the other hand, social sciences are much more fragmented: anthropology, communication, criminology, demography, economics and sociology can all be considered social sciences. Yet, there is no large scientific society that regroups researchers from these disciplines and that also publishes the various journals covering these different disciplines. There are, rather, many different associations for each discipline, which are often divided into specialities. Along these lines, topics in SSH are also more often local in scope—and thus much less international—which also lead to more decentralized—and thus smaller—scientific societies. As a consequence, these scientific societies did not have the means to adapt to the digital era and therefore were more likely to be acquired or have agreements with big commercial publishers for the publication of their journals. This is a clear shift from the traditional model of scholarly communication. As Lyman and Chodorow [25] put it:

“University presses and disciplinary associations were founded to disseminate research in the original cycle of scholarly communication. The faculty produced the work to be published; non-profit publishers organized the distribution of knowledge; the university library bought the published work at an artificially high price, as a subsidy for learned societies; and the faculty used this literature as the foundation for further research and teaching. […] However, over the past fifty years, as federal research funding has encouraged specialization, journal publishing has become commercialized, and some parts of the scientific and technical literature are now being monopolized by multinational publishing conglomerates.” (p. 89)

### The economics of scholarly publishing

**Fig 7:**
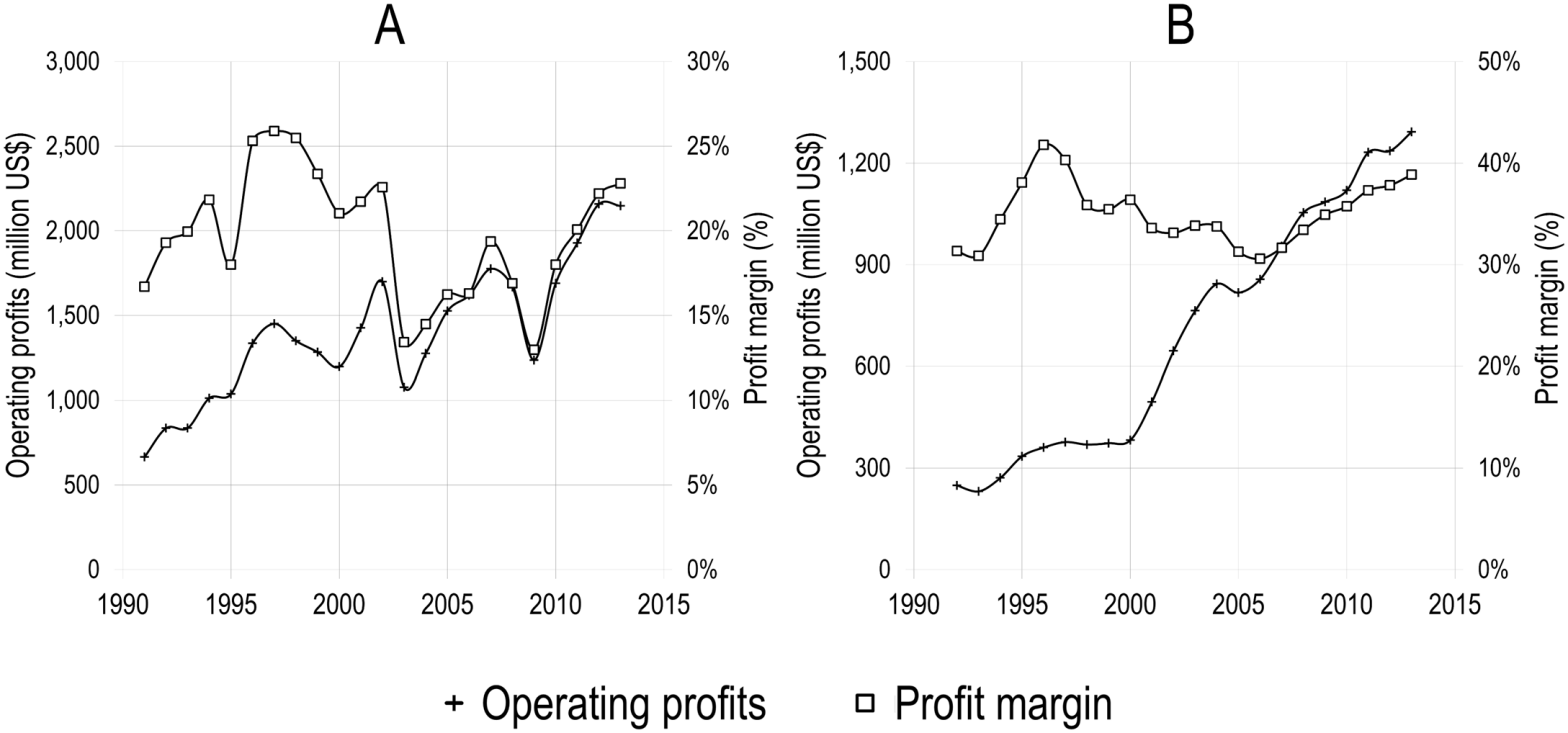
Operating profits (million USD) and profit margin of Reed-Elsevier as a whole (A) and of its Scientific, Technical & Medical division (B), 1991–2013. Compilation by the authors based on the annual reports of Reed-Elsevier. (http://www.reedelsevier.com/investorcentre/pages/home.aspx) Numbers for the Scientific, Technical & Medical division were only available in GBP; conversion to USD was performed using historical conversion rates from http://www.oanda.com.

As one might expect, the consolidation of the publishing industry led to an increase of the profits of publishers. Fig 7 presents, as an example, the evolution of Reed-Elsevier’s profits over the 1991–2013 period, for the firm taken as a whole as well as for its Scientific, Technical & Medical division. One can clearly see in Fig 7A that, between 1991 and 1997, both the profits and the profit margin increased steadily for the company as a whole. While profits more than doubled over that period—from 665M USD to 1,451M USD—profit margin also rose from 17% to 26%. Profit margins decreased, however, between 1998 and 2003, although profits remained relatively stable. Absolute profits as well as the profit margin then rose again, with the exception of the 2008–2009 period of economic crisis, resulting in profits reaching an all-time high of more than 2 billion USD in 2012 and 2013. The profit margin of the company’s Scientific, Technical & Medical division is even higher (Fig 7B). Moreover, its profits increased by a factor of almost 6 throughout the period, and never dropped below 30% from one year to another. The profit margin of this division never decreased below 30% during the period observed, and steadily increased from 30.6% to 38.9% between 2006 and 2013. Similarly high profit margins were obtained in 2012 by Springer Science+Business Media (35.0%, see: http://static.springer.com/sgw/documents/1412702/application/pdf/Annual_Report_2012_01.pdf), and in 2013 and John Wiley & Sons’ Scientific, Technical, Medical and Scholarly division (28.3%, see: http://www.wiley.com/legacy/about/corpnews/fy13_10kFINAL.pdf) and Taylor and Francis (35.7%, see: http://www.informa.com/Documents/Investor%20Relations/Annual%20Report%202013/Informa%20plc%20Annual%20Report%20Accounts%202013.pdf), putting them on a comparable level with Pfizer (42%), the Industrial & Commercial Bank of China (29%) and far above Hyundai Motors (10%), which comprise the most profitable drug, bank and auto companies among the top 10 biggest companies respectively, according to Forbes’ Global 2000 [30]. At a total revenue of 9.4 billion US dollars in 2011 [31], the majority of which were generated by a few publishing houses, the scientific journal publishing market faces oligopolistic conditions, where big players such as Elsevier, Springer, Taylor & Francis, Wiley-Blackwell and Wolters Kluwer determine annually increasing subscription rates that make up a considerable amount of research spending, leaving academic libraries with no other choice but to cancel subscriptions [20,32,33].

The possibility to increase profits in such an extreme fashion lies in the peculiarity of the economics of scholarly publishing. Unlike usual suppliers, authors provide their goods without financial compensation and consumers (i.e. readers) are isolated from the purchase. Because purchase and use are not directly linked, price fluctuations do not influence demand. Academic libraries, contributing 68% to 75% of journal publishing revenues [31], are atypical buyers because their purchases are mainly controlled by budgets. Regardless of their information needs, they have to manage with less as prices increase. Due to the publisher’s oligopoly, libraries are more or less helpless, for in scholarly publishing each product represents a unique value and cannot be replaced [19,20,33,34].

Scholarly publications themselves can be considered information goods with high fixed and low variable costs [35,36]. Regarding academic journals, fixed or first-copy costs comprise manuscript preparation, selection and reviewing as well as copy-editing and layout, writing of editorials, marketing, and salaries and rent, the two most substantial of which, manuscript writing and reviewing, are provided free of charge by the scholarly community [20]. In that sense and contrary to any other business, academic journals are an atypical information good, because publishers neither pay the provider of the primary good—authors of scholarly papers—nor for the quality control—peer review. On the publisher’s side, average first-copy costs of journal papers are estimated to range between 20 and 40 US dollars per page, depending on rejection rates [37]; [17], which neither explains open access publication fees as high as 5,000 $US (e.g., Cell Reports by Elsevier) nor hybrid journals, where publishers charge twice per article, i.e. the subscription and open access fees (e.g., Open Choice by Springer or Online Open by Sage Publications).

In addition, the Ingelfinger law, initiated by the publisher of the New England Journal of Medicine in 1969, prohibits authors from submitting their manuscript to more than one journal [38]. Although the law was initially created to protect the journal’s revenue streams and has become largely obsolete through electronic publishing [39], it is still a universal rule in academic journal publishing, often enforced by copyright transfer agreements. Hence, each journal has the monopoly on the scientific content of papers it publishes: paper A published in journal Y is not an alternative to paper B published in journal Z [11]. In other words, access to paper A does not replace access to paper B, both papers being complementary to each other.

Variable costs of academic journals are paid by the publisher and, as long as journals were printed and distributed physically, these costs were sizeable. In the print era, publishers had to typeset the manuscripts, print copies of journals, and send them to various subscribers. Hence, each time an issue was printed, sent and sold, another copy had to be printed to be sent and sold. However, with the advent of electronic publishing, these costs became marginal. The digital era exacerbated this trend and increased the potential revenues of publishers. While, in economic terms, printed journals can be considered as rival goods—goods that cannot be owned simultaneously by two individuals—online journals are non-rival goods [40]: a single journal issue that has been uploaded by the publisher on the journal’s website can be accessed by many researchers from many universities at the same time. The publisher does not have to upload or produce an additional copy each time a paper is accessed on the server as it can be duplicated *ad infinitum*, which in turn reduces the marginal cost of additional subscriptions to 0. In a system where the marginal cost of goods reaches 0, their cost becomes arbitrary and depends merely on how badly they are needed, as well as by the purchasing power of those who need them. In addition, costs are strongly influenced by the power relations between the buyer and seller, i.e. publishers and academic libraries. In such a system, any price is good for the seller, as the additional unit sold is pure profit. All these factors explain the different and often irrational big deals made between publishers and subscribers, with university libraries subscribing to a publisher’s entire set or large bundle of journals regardless of their specific needs [41]. Through these big deals, university researchers have been accustomed to, for almost 20 years, having access to an increasingly large proportion of the scientific literature published, which makes it very difficult for university libraries today to cancel subscriptions and negotiate out of big deals with publishers to optimize their collections and meet budget restrictions.

### General conclusions

Since the creation of scientific journals 350 years ago, large commercial publishing houses have increased their control of the science system. The proportion of the scientific output published in journals under their ownership has risen steadily over the past 40 years, and even more so since the advent of the digital era. The value added, however, has not followed a similar trend. While one could argue that their role of typesetting, printing, and diffusion were central in the print world [20,7], the ease with which these function can be fulfilled—or are no longer necessary—in the electronic world makes one wonder: what do we need publishers for? What is it that they provide that is so *essential* to the scientific community that we collectively agree to devote an increasingly large proportion of our universities budgets to them? Of course, most journals rely on publishers’ systems to handle and review the manuscripts; however, while these systems facilitate the process, it is the researchers as part of the scientific community who perform peer review. Hence, this essential step of quality control is not a value added by the publishers but by the scientific community itself.

Thus, it is up to the scientific community to change the system in a similar fashion and in parallel to the open access and open science movements. And, indeed, the scientific community has started to react to and protest against the exploitative behaviour of the major for-profit publishers. In 2012, the “Cost of Knowledge” (http://thecostofknowledge.com/) campaign started by Cambridge mathematician and Fields Medalist Timothy Gowers asked researchers to protest against Elsevier’s business model through a boycott against its journals by ceasing to submit to, edit and referee them. Started by a blogpost, the boycott was later termed the beginning of an “Academic Spring” [42,43]. Several university libraries, including large and renowned universities such as the University of California [44] and Harvard [45], stopped negotiations and threatened to boycott major for-profit publishers, while other universities—such as the University of Konstanz—simply cancelled all Elsevier subscriptions as they were neither able nor willing to keep up with their aggressive pricing policy: 30% increase over five years [46,47].

But these are exceptions. Unfortunately, researchers are still dependent on one essentially symbolic function of publishers, which is to allocate academic capital, thereby explaining why the scientific community is so dependent on ‘The Most Profitable Obsolete Technology in History’ [48]. Young researchers need to publish in prestigious journals to gain tenure, while older researchers need to do the same in order to keep their grants, and, in this environment, publishing in a high impact Elsevier or Springer journal is what ‘counts’. In this general context, the negative effect of various bibliometric indicators in the evaluation of individual researchers cannot be understated. The counting of papers indexed by large-scale bibliometric databases—which mainly cover journals published by commercial publishers, as we have seen in this paper—creates a strong incentive for researchers to publish in these journals, and thus reinforces the control of commercial publishers on the scientific community.
